# CG200745, a Novel HDAC Inhibitor, Attenuates Kidney Fibrosis in a Murine Model of Alport Syndrome

**DOI:** 10.3390/ijms21041473

**Published:** 2020-02-21

**Authors:** Sang Heon Suh, Hong Sang Choi, Chang Seong Kim, In Jin Kim, Hyunju Cha, Joong Myung Cho, Seong Kwon Ma, Soo Wan Kim, Eun Hui Bae

**Affiliations:** 1 Department of Internal Medicine, Chonnam National University Medical School, Gwangju 61469, Korea; medssh1984@gmail.com (S.H.S.); hongsang38@hanmail.net (H.S.C.); laminion@hanmail.net (C.S.K.); remon9127@hanmail.net (I.J.K.); drmsk@hanmail.net (S.K.M.); 2 Department of Internal Medicine, Chonnam National University Hospital, Gwangju 61469, Korea; 3 Crystal Genomics, Inc., 5 F, Bldg A, Korea Bio Park, Seongnam 13488, Korea; hjcha@cgxinc.com (H.C.); jmcho@cgxinc.com (J.M.C.)

**Keywords:** CG200745, *Col4a3* knockout, Alport syndrome, histone deacetylase

## Abstract

Histone deacetylases have been a target of therapy for organ fibrosis. Here, we report the protective effect of CG200745 (CG), a novel histone deacetylase inhibitor, on tubulointerstitial fibrosis in *Col4a3*^−/−^ mice, a murine model of Alport syndrome. Morphological analyses revealed CG treatment markedly alleviated kidney fibrosis in *Col4a3*^−/−^ mice at the age of 7 weeks. CG prevented the activation of transforming growth factor β (TGFβ) and its downstream SMAD signaling in the kidney of *Col4a3*^−/−^ mice. As critical upstream regulators of TGFβ signaling, immunoblotting of whole kidney lysate of *Col4a3*^−/−^ mice reveled that intra-renal renin–angiotensin system (RAS) was activated with concurrent upregulation of inflammation and apoptosis, which were effectively suppressed by CG treatment. CG suppressed both activation of RAS and up-regulation of TGFβ signals in angiotensin II-stimulated HK-2 cells, a human kidney proximal tubular epithelial cell line. CG inhibited activation of TGFβ-driven signals and fibrosis in NRK-49F cells, a rat kidney fibroblast cell line, under angiotensin II-rich conditions. Collectively, CG was found to be effective both in proximal tubular epithelial cells by inhibiting local RAS and TGFβ signaling activation, as well as in fibroblasts by blocking their transition to myofibroblasts, attenuating renal fibrosis in a murine model of Alport syndrome.

## 1. Introduction

Alport syndrome (AS) is a hereditary type IV collagen disorder resulting from the mutations in the gene *COL4A3/4/5*, which encodes α3/α4/α5 type IV collagen chains [1,2], with primary defects in postnatal maturation of glomerular basement membrane (GBM) [3,4]. The use of *Col4a3*-deficient (*Col4a3*^−/−^) mice has been an experimental model of human AS, which causes the development of proteinuria as early as the age of postnatal 6 weeks, with progressive glomerulonephritis and tubulointerstitial scarring, and leads to death with end-stage renal disease at the age of approximately 14 weeks [5]. Although the pathology begins at the glomeruli, fibrosis commonly progresses in the tubulointerstitium as well as in the glomeruli of *Col4a3*^−/−^ mice [5]. It is worthy to note that tubulointerstitial fibrosis is both a key feature of disease progression and a therapeutic target of AS, as a previous report demonstrated that, despite the persistent ultrastructural defect in GBM, a pre-emptive pharmacologic intervention attenuated inflammation and tubulointerstitial fibrosis, and subsequently delayed the onset of end-stage renal disease in *Col4a3*^−/−^ mice [6].

**Figure 1:**
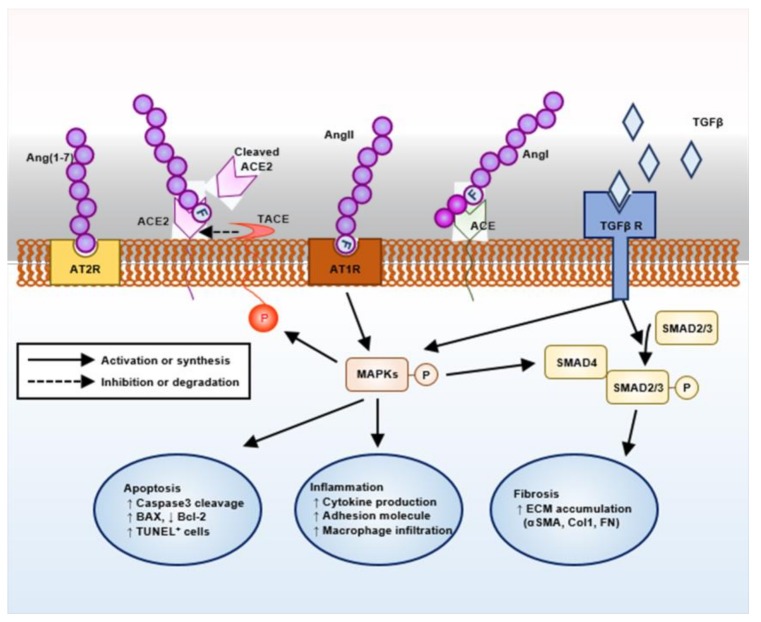
Schematic overview on the intra-renal renin–angiotensin system (RAS) machinery and its potential action mechanism.

Evidence now suggests the pivotal role of the renin–angiotensin system (RAS) in the pathogenesis of AS (Figure 1). Targeting RAS with angiotensin-converting enzyme inhibitors or angiotensin II receptor blockers (ARBs) has been proven to protect kidney in human AS patients as well as in animal models [7,8,9]. Angiotensin converting enzyme (ACE) 2 was originally identified as a human homology of ACE, and generates angiotensin-(1–9) from Ang (angiotensin) I or degrades Ang II to the angiotensin-(1–7) (Ang-(1–7)) [10], although the catalytic activity of ACE2 for Ang II is 400-fold greater for Ang I [11]. As Ang-(1–7) is a vasodilator generated as a result of Ang II degradation, Ang-(1–7) and its related enzyme ACE2 have been expected to counter-regulate the classical RAS pathway involving ACE and Ang II [12,13]. Although Ang II mainly acts via angiotensin type 1 receptor (AT1R) leading to target organ damage [14,15], Ang (1–7) can act via both mitochondrial assembly 1 proto-oncogene protein (MAS), a G-protein-coupled receptor, and angiotensin type 2 receptor (AT2R), where both vasoprotective and atheroprotective effects mediated by Ang (1–7) can be abolished by point mutation or chemical inhibition of AT2R [16]. Ang II induces proteolytic cleavage of ACE2 by promoting TNF-α-converting enzyme (TACE) activity, where Ang II facilitates positive feedback loop by loss of its negative regulator, ACE2 [17]. Indeed, conventional knockout of ACE2 is associated with age-dependent development of glomerulosclerosis and albuminuria, as well as the exacerbation of diabetic kidney injury in mice [18]. Despite these intensive studies, our current strategy to activate an ‘alternative’ ACE2–Ang-(1–7) pathway is still experimental without clinically available materials.

Histone deacetylase (HDAC) inhibitors, which inhibit histone deacetylases to remove the acetyl moiety from histone tails, and thereby promote histone acetylation [19], was originally introduced as an anti-cancer therapeutic [20,21], and also effectively prevents organ fibrosis in various experimental models [19]. Pathological cardiac hypertrophy and fibrosis was blocked in deoxycorticosterone acetate-induced [22] or spontaneously hypertensive rats [23]. Their anti-fibrotic potential has also been implicated in animal models of diseases involving kidney [24,25] as well as liver and lung [26,27]. CG200745 ((E)-N(1)-(3-(dimethylamino)propyl)-N(8)-hydroxy-2-((naphthalene-1-loxy)methyl)oct-2-enediamide, CG) is a novel HDAC inhibitor that blocks class I and II HDACs, with a stronger potency to acetylation effect than vorinostat, another pan-HDAC inhibitor [21,28], and was recently successfully evaluated for its tolerability in human subjects [29]. Inspired by its safety profile in human study, we reported that CG alleviates the renal fibrosis in a unilateral ureteral obstruction animal model, a prototype of human obstructive uropathy [25]. In the present study, we examined whether CG could ameliorate the disease progression in a murine model of AS, where the organ fibrosis is primarily driven by activation of local RAS. We found that CG modifies molecular pathways of renal fibrosis both in tubular epithelial cells and in fibroblasts. Intriguingly, CG preserved ACE2 expression in Ang II-stimulated tubular epithelial cells, aiming at a critical pathways in the pathogenesis of AS.

## 2. Results

### 2.1. CG Alleviated Kidney Fibrosis in Col4a3^−/−^ Mice

To assess the effect of CG treatment on the kidney function in *Col4a3*^−/−^ mice, we measured neutrophil gelatinase-associated lipocalin (NGAL) level in urine as well as body and kidney weights (Table 1). Compared to wild type (WT) mice, body weight was significantly decreased in *Col4a3*^−/−^ mice, regardless of CG treatment. Kidney weight was not different among the three groups. Accordingly, the ratio of kidney weight to body weight was significantly increased in *Col4a3*^−/−^ mice, suggesting tissue inflammation, which was moderately recovered by CG treatment. CG also partially normalized increased urine NGAL level in *Col4a3*^−/−^ mice, indicating its protective role in the tubulointerstitial injury.

**Figure 2:**
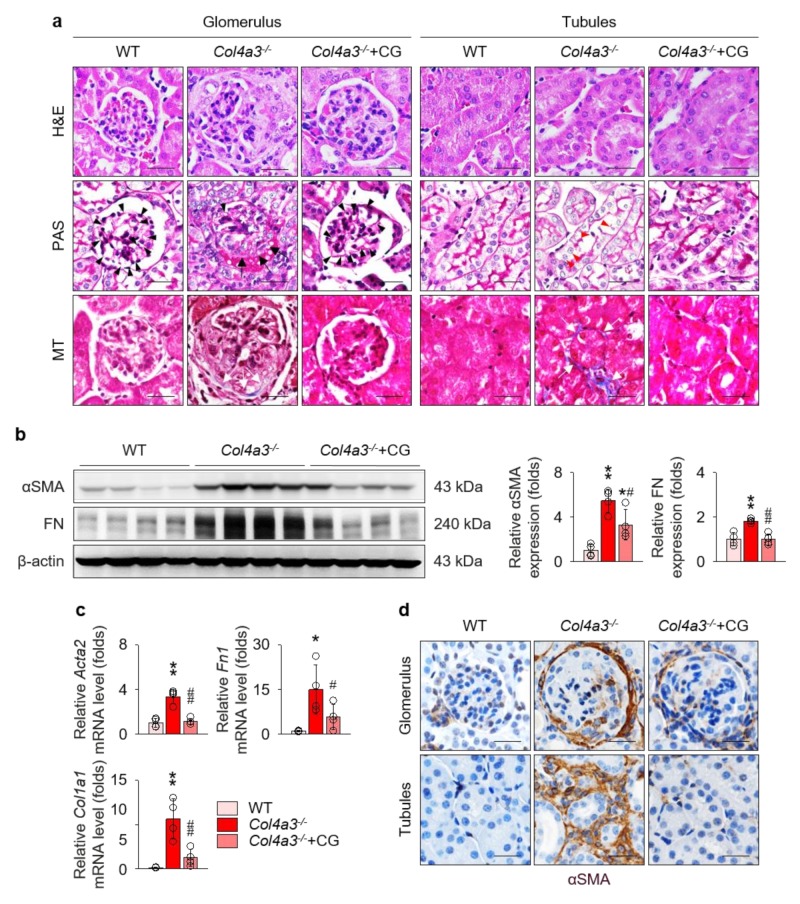
CG alleviated kidney fibrosis in *Col4a3*^−/−^ mice. (**a**) Tissue morphology of kidney from WT, *Col4a3*^−/−^, and *Col4a3*^−/−^+CG mice. Images from glomerulus (left) and tubulointerstitium (right) are presented. H&E, hematoxylin and eosin staining; PAS, periodic acid Schiff staining; MT, Masson’s trichrome staining. Note that, contrary to kidneys from the other groups, glomerular capillary lumens were barely patent (arrowheads) with shrinkage of mesangium (white asterisk) in *Col4a3*^−/−^ mice. In *Col4a3*^−/−^ mice, crescents were packing the Bowman’s space (arrows). Detachment of tubular epithelial cells from basement membrane (red arrowheads) and collagen deposits (white arrows) are also indicated. Scale bars, 50 μm. (**b**,**c**) Comparison of expression level for fibrosis markers determined by immunoblotting (**b**) and qPCR (**c**) from the kidney of WT, *Col4a3*^−/−^, and *Col4a3*^−/−^+CG mice (*n* = 4 mice/group). β-actin for immunoblotting and glyceraldehyde 3-phosphate dehydrogenase (GAPDH) for qPCR were used as the endogenous controls. (**d**) Representative images of immunohistochemical staining for α smooth muscle actin (αSMA; stained in brown) in the kidney of WT, *Col4a3*^−/−^, and *Col4a3*^−/−^+CG mice. Images from glomeruli (upper) and tubules (lower) are presented. Scale bars, 50 μm. *p < 0.05, **p < 0.01 vs. WT mice; ^#^*p* < 0.05, ^##^*p* < 0.01 vs. *Col4a3*^−/−^ mice by one-way ANOVA with Newman–Keuls multiple comparison test.

To compare the tissue morphology, a series of histochemical staining, including hematoxylin and eosin, periodic acid Schiff, and Masson’s trichrome staining, were evaluated in the kidney of WT and *Col4a3*^−/−^ mice (Figure 2a). Contrary to the glomeruli of WT mice, fibrotic crescents were observed in the Bowman’s space of *Col4a3*^−/−^ mice. With shrinkage of mesangium, capillary loops were significantly choked in *Col4a3*^−/−^ mice. In tubulointerstitial compartment, tubular necrosis characterized by detachment of tubular epithelial cells from basement membranes was evident in *Col4a3*^−/−^ mice, with obvious collagen deposit in interstitial space. CG treatment considerably prevented such morphological abnormalities of glomeruli and tubulointerstitium in the histochemical staining. To characterize kidney fibrosis at the molecular level, fibrosis markers were compared among the group. Immunoblotting (Figure 2b) revealed up-regulation of α smooth muscle actin (αSMA) and fibronectin, whereas quantitative polymerase chain reaction (qPCR) (Figure 2c) demonstrated upregulation of collagen type 1 as well as αSMA and fibronectin in the whole kidney lysate of *Col4a3*^−/−^ mice. Up-regulation of those fibrosis markers was substantially aborted by CG administration. Anatomically, αSMA expression was up-regulated both in glomeruli and in peritubular interstitium, but not in the tubular epithelial cells, of *Col4a3*^−/−^ mice (Figure 2d), which was significantly counteracted by CG treatment. Taken together, these data suggest that CG alleviates kidney fibrosis in *Col4a3*^−/−^ mice.

### 2.2. Up-Regulation of TGFβ in the Kidney of Col4a3^−/−^ Mice Was Attenuated by CG

**Figure 3:**
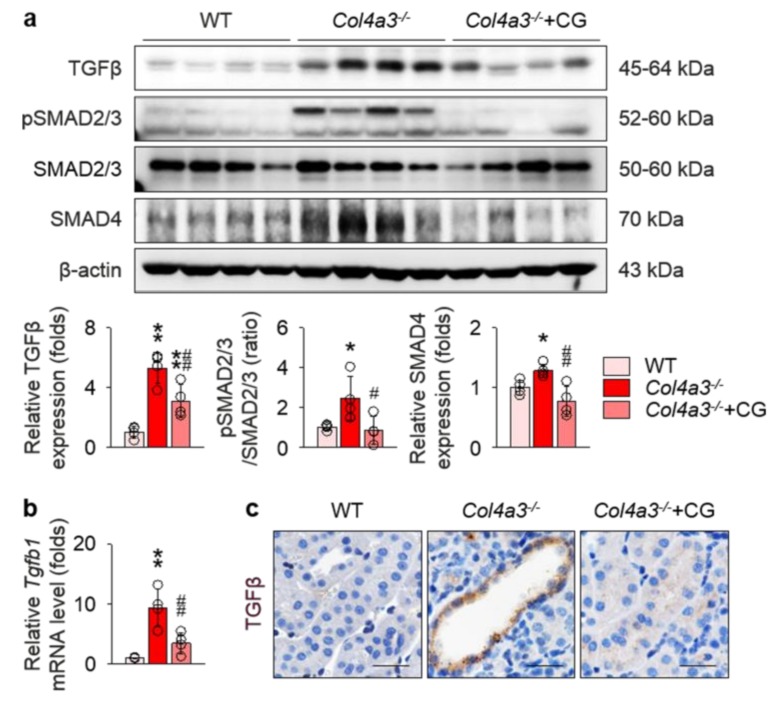
CG attenuated activation of transforming growth factor β (TGFβ) signals in *Col4a3*^−/−^ mice. (**a**) Comparison of protein expression level TGFβ and its downstream signals determined by immunoblotting from the kidney of WT, *Col4a3*^−/−^, and *Col4a3*^−/−^+CG mice (*n* = 4 mice/group). β-actin was used as the endogenous control. (**b**) Comparison of mRNA expression level *Tgfb1* determined by qPCR from the kidney WT, *Col4a3*^−/−^, and *Col4a3*^−/−^+CG mice (*n* = 4 mice/group). β-actin was used as the endogenous control. (**c**) Representative images of immunohistochemical staining for TGFβ (stained in brown) in the renal cortex of WT, *Col4a3*^−/−^, and *Col4a3*^−/−^+CG mice. Scale bars, 50 μm. *p < 0.05, **p < 0.01 vs. WT mice; ^#^*p* < 0.05, ^##^*p* < 0.01 vs. *Col4a3*^−/−^ mice by one-way ANOVA with Newman–Keuls multiple comparison test.

To address underlying molecular events relevant to tissue fibrosis in *Col4a3*^−/−^ mice, we focused on the transforming growth factor β (TGFβ) pathway, one of the key mechanisms in the progression of tubulointerstitial fibrosis. As expected, immunoblotting (Figure 3a) and qPCR (Figure 3b) on the whole kidney lysate revealed up-regulation of TGFβ in *Col4a3*^−/−^ mice, which was significantly reduced by CG administration. Enhanced phosphorylation of SMAD2/3 and increased expression of SMAD4, a canonical downstream of TGFβ receptor, was also significantly attenuated by CG treatment (Figure 3a). To specify the anatomical site or cellular component with TGFβ up-regulation, tissues were examined with immunohistochemical staining (Figure 3c). Intense TGFβ signals were observed in the tubulointerstitial compartment, especially in the epithelial cells of markedly dilated, atrophied tubules of *Col4a3*^−/−^ mice, compared to the other two groups. Collectively, our findings suggest CG efficiently blocks up-regulation of TGFβ signaling in the kidney of *Col4a3*^−/−^ mice.

### 2.3. Up-Regulation of Intra-Renal RAS in Col4a3^−/−^ Mice Was Counter-Regulated by CG, with Preservation of ACE2 Expression

**Figure 4:**
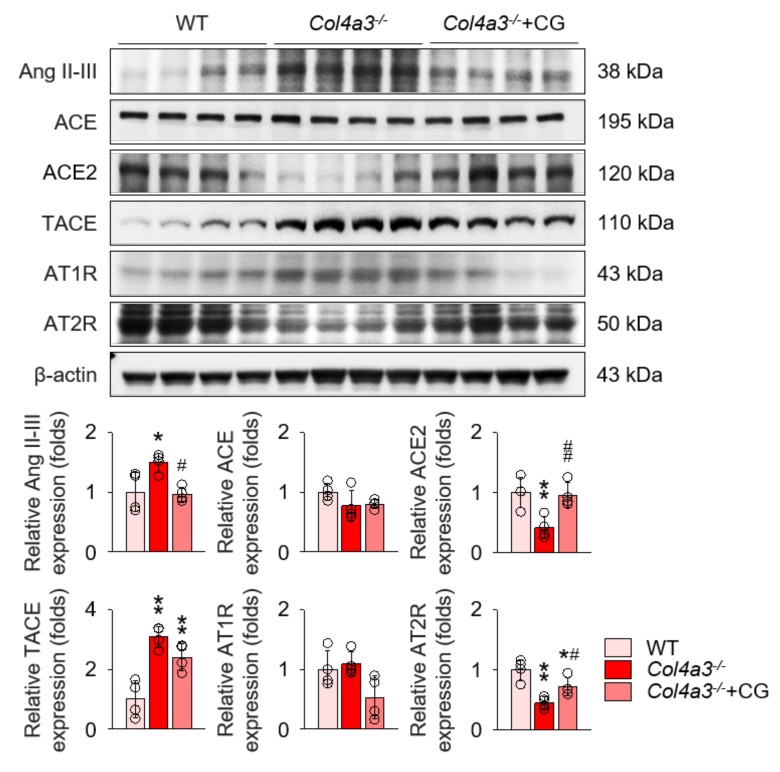
CG inhibited RAS activation in *Col4a3*^−/−^ mice. Comparison of protein expression level of RAS components determined by immunoblotting from the kidney of WT, *Col4a3*^−/−^, and *Col4a3*^−/−^+CG mice (*n* = 4 mice/group). β-actin was used as the endogenous control. *p < 0.05, **p < 0.01 vs. WT mice; ^#^*p* < 0.05, ^##^*p* < 0.01 vs. *Col4a3*^−/−^ mice by one-way ANOVA with Newman–Keuls multiple comparison test.

**Figure 5:**
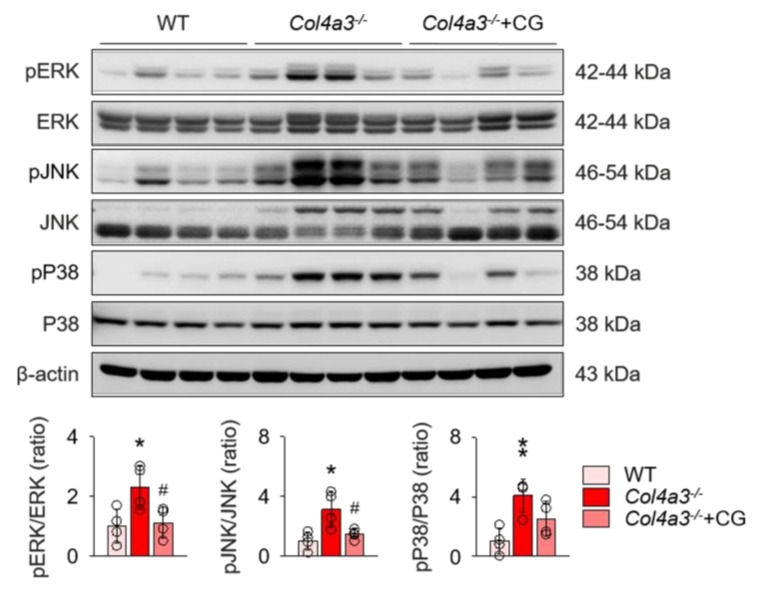
Enhanced phosphorylation of ERK and JNK in *Col4a3*^−/−^ mice was suppressed by CG. Comparison of protein expression level of mitogen-activated protein kinases (MAPKs) determined by immunoblotting from the kidney of WT, *Col4a3*^−/−^, and *Col4a3*^−/−^+CG mice (*n* = 4 mice/group). β-actin was used as the endogenous control. *p < 0.05, **p < 0.01 vs. WT mice; ^#^*p* < 0.05 vs. *Col4a3*^−/−^ mice by one-way ANOVA with Newman–Keuls multiple comparison test.

To unveil the upstream of TGFβ signaling that is blocked by CG, we investigated the alterations of intra-renal RAS components in *Col4a3*^−/−^ mice by CG treatment. Immunoblotting of whole kidney lysate (Figure 4) demonstrated that, compared to WT and CG-treated groups, tissue Ang II-III level was distinctively increased in *Col4a3*^−/−^ mice, although tissue expression of ACE was not different among the groups. Instead, ACE2 and TACE were significantly down-regulated and up-regulated, respectively, in *Col4a3*^−/−^ mice. AT2R was down-regulated in *Col4a3*^−/−^ mice, whereas Ang II type 1 receptor (AT1R) expression remained unaffected. CG significantly prevented up-regulation of tissue Ang II-III level and down-regulation of ACE2 and AT2R in *Col4a3*^−/−^ mice, although up-regulation of TACE was not reversed by CG. Thus, these results collectively indicate that CG effectively counter-balances the activation of intra-renal RAS in *Col4a3*^−/−^ mice. To clarify the link between intra-renal RAS and TGFβ signaling, we examined mitogen-activated protein kinases (MAPKs), which have been suggested to relay the signal originated from AT1R to TGFβ transcription [30]. Immunoblotting of whole kidney lysate (Figure 5) showed that phosphorylation of extracellular signal-regulated kinases (ERK), c-Jun N-terminal kinases (JNK), and p38 mitogen-activated protein kinases (P38) was enhanced in *Col4a3*^−/−^ mice, where CG blocked phosphorylation of ERK and JNK, but not P38, in *Col4a3*^−/−^ mice. Accordingly, it was suggested that both activation of classical RAS pathway and conduction of its downstream signal to TGFβ transcription in *Col4a3*^−/−^ mice were suppressed by CG treatment.

### 2.4. CG Prevented Apoptosis and Inflammation in the Kidney of Col4a3^−/−^ Mice

**Figure 6:**
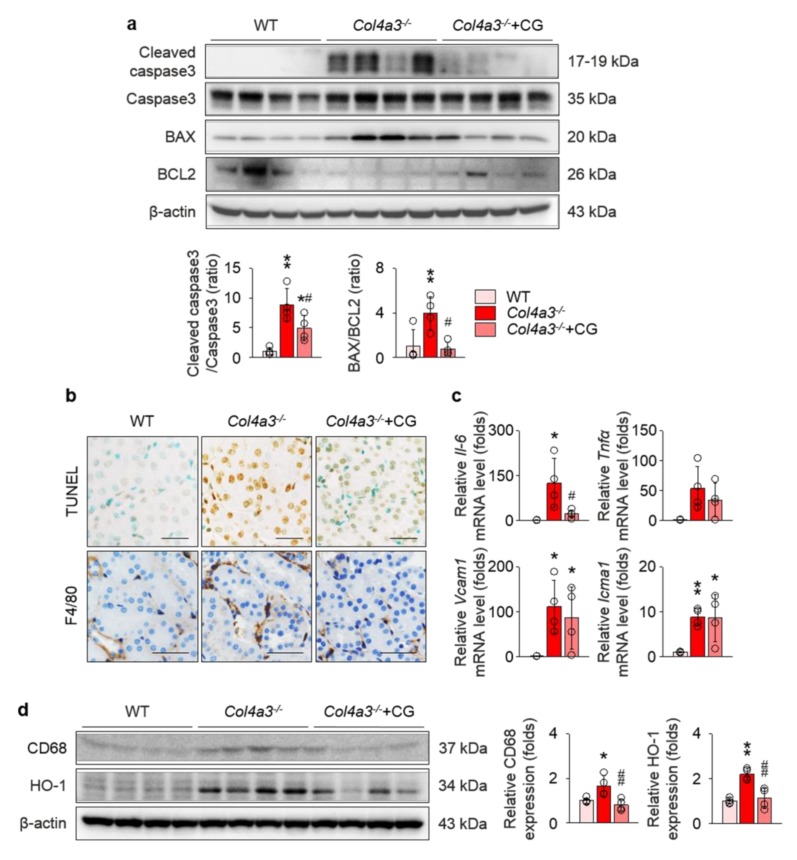
CG alleviated apoptosis and inflammation in the kidney of *Col4a3*^−/−^ mice. (**a**) Comparison of protein expression level for molecules related to apoptosis determined by immunoblotting from the kidney of WT, *Col4a3*^−/−^, and *Col4a3*^−/−^+CG mice (*n* = 4 mice/group). β-actin was used as the endogenous control. (**b**) Representative images of terminal deoxynucleotidyl transferase dUTP nick end labeling (TUNEL; upper, stained in brown) and immunohistochemical staining for F4/80 (lower, stained in brown) in the kidney of WT, *Col4a3*^−/−^, and *Col4a3*^−/−^+CG mice. (**c**) Comparison of mRNA expression level for inflammatory markers determined by qPCR from the kidney of WT, *Col4a3*^−/−^, and *Col4a3*^−/−^+CG mice (*n* = 4 mice/group). GAPDH was used as the endogenous control. (**d**) Comparison of protein expression level for CD68 and heme oxygenase 1 (HO-1) determined by immunoblotting from the kidney of WT, *Col4a3*^−/−^, and *Col4a3*^−/−^+CG mice (*n* = 4 mice/group). β-actin was used as the endogenous control. Scale bars, 50 μm. *p < 0.05, **p < 0.01 vs. WT mice; ^#^*p* < 0.05, ^##^*p* < 0.01 vs. *Col4a3*^−/−^ mice by one-way ANOVA with Newman–Keuls multiple comparison test.

To reveal the consequences following MAPK activation that may accelerate kidney fibrosis, we investigated the alterations of apoptotic and inflammatory processes in the kidney of *Col4a3*^−/−^ mice, as these processes are medicated by activation of MAPKs [31,32]. Cleavage of caspase-3 and Bcl-2-associated X protein (BAX/B-cell lymphoma 2 (BCL2) ratio were enhanced in the immunoblotting of whole kidney lysate from *Col4a3*^−/−^ mice (Figure 6a). Morphologically, terminal deoxynucleotidyl transferase deoxyuridine Triphosphate (dUTP) nick end labeling (TUNEL)-positive tubular epithelial cells in renal cortices were considerably increased in *Col4a3*^−/−^ mice (Figure 6b), collectively suggesting that, compared to WT mice, the apoptotic process is more active in the kidney, especially in the cortical tubular epithelial cells, of *Col4a3*^−/−^ mice. In addition, immunohistochemical staining of F4/80 visualized marked infiltration of macrophages in the interstitial space of *Col4a3*^−/−^ mice (Figure 6b), implying a concomitant inflammatory process. Further, we tested a series of molecules that are known to be upregulated during the inflammatory process [9,24,25]. Indeed, qPCR using whole kidney lysate (Figure 6c) demonstrated significant up-regulation of inflammatory cytokines and adhesion molecules, such as interleukin-6, vascular cell adhesion molecule 1, and intercellular adhesion molecule 1. Immunoblotting of whole kidney lysate (Figure 6d) demonstrated up-regulation of CD68 and hemoxygenase-1 in *Col4a3*^−/−^ mice, indicating underlying tissue inflammation. CG treatment, at large, attenuated alterations in apoptotic and inflammatory processes of *Col4a3*^−/−^ mouse kidney (Figure 6a–d).

### 2.5. CG Attenuated RAS Activation and TGFβ up-Regulation in HK-2 Cells Under Ang II-Rich Condition

**Figure 7:**
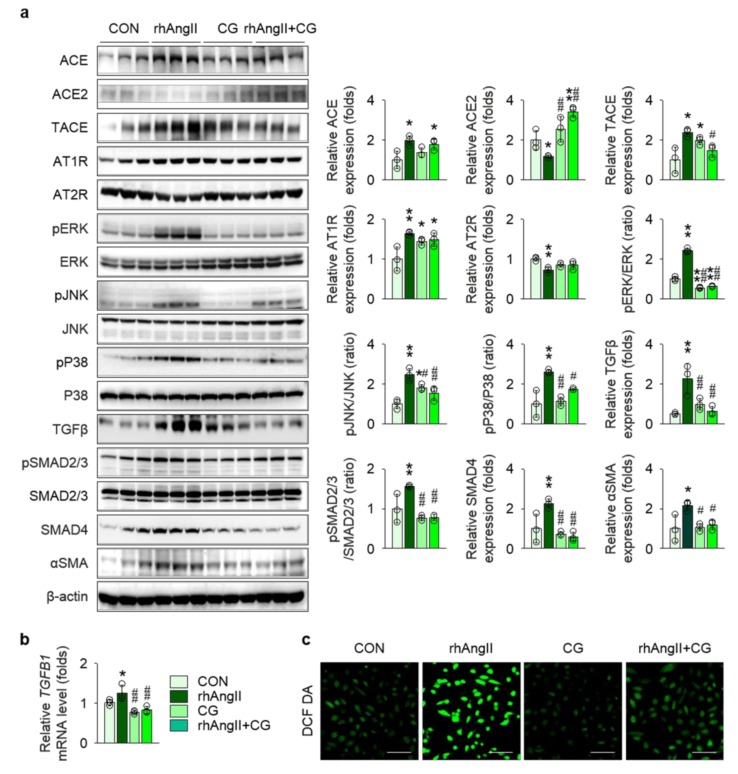
CG abrogated activation of RAS and TGFβ signals in Ang II-stimulated HK-2 cells. (**a**) Comparison of protein expression level for RAS components, MAPKs, and TGFβ and its downstream molecules in HK-2 after stimulation with vehicles or recombinant human Ang II (rhAng II) with or without co-treatment of CG (*n* = 3/group). β-actin was used as the endogenous control. Data are representative more than three independent experiments. (**b**) Comparison of mRNA expression level for *TGFB1* determined by qPCR in HK-2 after stimulation with vehicles or recombinant human Ang II (rhAng II) with or without co-treatment of CG (*n* = 3/group). GAPDH was used as the endogenous control. (**c**) Representative images of 5,6-chloromethyl-2′,7′-dichlorodihydrofluorescein diacetate (DCF DA) staining in HK-2 after stimulation with vehicles or recombinant human Ang II (rhAng II) with or without co-treatment of CG. Data are representative more than three independent experiments. Scale bars, 200 μm. *p < 0.05, **p < 0.01 vs. control cells (CON); ^#^*p* < 0.05, ^##^*p* < 0.01 vs. rhAng II-treated cells by one-way ANOVA with Newman–Keuls multiple comparison test.

To precisely validate the anatomical site of action, we examined the effect of CG treatment in Ang II-stimulated HK-2 cells, a human kidney proximal tubular epithelial cell line, as the proximal nephron segment has been known to be a major apparatus for local RAS machinery. CG partially, but not completely, attenuated RAS activation in Ang II-stimulated HK-2 cells, as Ang II-treatment in HK-2 cells up-regulated ACE, TACE, and AT1R and down-regulated ACE2 and AT2R, suggesting a positive feedback loop of intra-renal RAS activation, whereas CG reversed some of the Ang II-induced alterations, such as ACE2 and TACE (Figure 7a). Ang II stimulation in HK-2 enhanced phosphorylation of ERK, JNK, and P38, all of which were significantly blocked by CG treatment. Most importantly, relevant to tissue fibrosis, CG substantially attenuated Ang II-induced up-regulation of TGFβ more than twofold in immunoblotting (Figure 7a) and to a lesser degree in qPCR (Figure 7b). Consequently, its downstream pathways, SMAD2/3 phosphorylation and SMAD4 expression, in Ang II-stimulated HK-2 cells were aborted by CG treatment. As tubular epithelial cells contribute to tissue fibrosis via epithelial-to-mesenchymal transition (EMT), αSMA expression was compared between the groups, where CG inhibited Ang II-induced EMT in HK-2 cells. Level of intracellular and mitochondrial reactive oxygen species assessed using 5,6-chloromethyl-2′,7′-dichlorodihydrofluorescein diacetate demonstrated that CG also reduced oxidative stress in Ang II-stimulated HK-2 cells (Figure 7c), implying the additional mechanism for amelioration of tissue inflammation in *Col4a3*^−/−^ mice with CG treatment. CG200745 neither promoted the apoptosis in HK-2 cells nor prevented apoptosis in Ang II-stimulated HK-2 cells (Figure S1).

### 2.6. CG Directly Inhibited Fibrotic Transition of Ang II-Stimulated Renal Fibroblast

**Figure 8:**
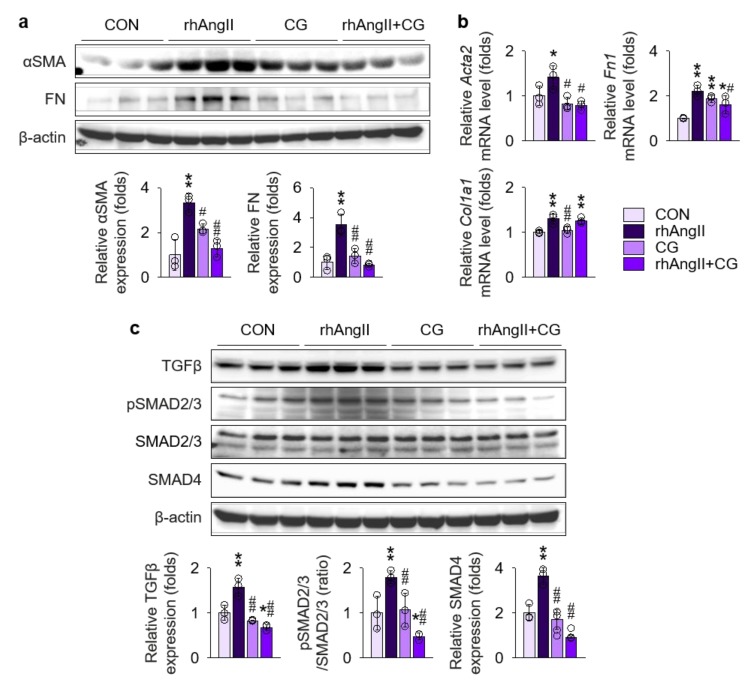
CG directly targeted activation of TGFβ signals in Ang II-stimulated NRK-49F cells. (**a**) Comparison of protein expression level for TGFβ and its downstream molecules in NRK-49F after stimulation with vehicles or recombinant human Ang II (rhAng II) with or without co-treatment of CG (*n* = 3/group). β-actin was used as the endogenous control. Data are representative more than three independent experiments. (**b**,**c**) Comparison of expression level for fibrosis markers determined by immunoblotting (**b**) and qPCR (**c**) in NRK-49F after stimulation with vehicles or recombinant human Ang II (rhAng II) with or without co-treatment of CG (*n* = 3/group). β-actin for immunoblotting and GAPDH for qPCR were used as the endogenous controls, respectively. *p < 0.05, **p < 0.01 vs. control cells (CON); ^#^*p* < 0.05, ^##^*p* < 0.01 vs. rhAng II-treated cells by one-way ANOVA with Newman–Keuls multiple comparison test.

To further test the direct effect of CG on the renal fibroblast under Ang II-rich condition, Ang II was treated to NRK-49F cells, a rat kidney fibroblast cell line, with or without co-treatment with CG (Figure 8), as fibroblasts are the major cellular origin of myofibroblasts [33]. Phenotypically, immunoblotting (Figure 8a) and qPCR (Figure 8b) of fibrosis markers demonstrated that CG blocks fibrotic transition of Ang II-stimulated NRK-49F cells. Mechanistically, we investigated TGFβ signals in NRK-49F treated with Ang II. TGFβ was significantly up-regulated in NRK-49F cells with Ang II treatment in the immunoblotting (Figure 8c), although the magnitude of increment was lesser (less than 2 folds) than in HK-2 cells (more than 2 folds in Figure 7a), which in turn activated SMAD2/3 phosphorylation and SMAD4 expression. Co-treatment with CG effectively suppressed both up-regulation of TGFβ and activation of its downstream signals in Ang II-stimulated NRK-49F cells. These results suggest that CG also directly targets activated renal fibroblasts under AngII-rich environments to delay the organ fibrosis.

## 3. Discussion

In this study, we demonstrated that CG ameliorates kidney fibrosis in *Col4a3*^−/−^ mice, a murine model of AS, where activation of local RAS is a core pathomechanism. We proved that CG inhibits molecular pathways of renal fibrosis in Ang II-stimulated tubular epithelial cells, with preservation of ACE2 expression in tubular epithelial cells and with suppression of TGFβ expression. Importantly, CG directly attenuated the transition of quiescent fibroblast to activated myofibroblast under Ang II-rich condition, suggesting its therapeutic potential against the final step of organ fibrosis.

Proximal tubule (PT) epithelial cells are the cornerstone of intra-renal RAS. PT epithelial cells uptake major components of RAS in the system circulation, such as angiotensinogen, renin, Ang I, and Ang II, which freely pass through the glomerular filtration barrier, in a manner dependent on AT1R [34,35] and/or megalin [36]. Inversely, systemic infusion of Ang II stimulates angiotensinogen expression in PT epithelial cells [37,38]. Moreover, apical expression of ACE in PT is dependent on megalin, where expression of ACE2, instead of ACE, becomes dominant with conditional deletion of megalin [36]. We here demonstrated that CG effectively counter-regulated the up-regulation of ACE in Ang II-stimulated HK-2 cells (Figure 7), with concurrent preservation of ACE2 expression, followed by blockade of MAPK phosphorylation, which is a crucial downstream pathway of RAS activation [39]. It is quite promising in that, although intra-renal RAS activation is one of the characteristics of AS, it is also prominently observed in the other kidney disease models, such as diabetic nephropathy [40]. It should further be elucidated whether the effect of CG in vitro could effectively work in the other animal model with intra-renal RAS activation.

Considering the positive feedback loop between intra-renal RAS activation and TGFβ signaling [9], our results from HK-2 cells (Figure 7) indicate that CG blocks the initiating process of the tubulointerstitial fibrosis in *Col4a3*^−/−^ mice. Nevertheless, this does not necessarily mean that CG directly alleviates the kidney fibrosis in *Col4a3*^−/−^ mice, as various cell populations contribute to the renal fibrosis, albeit epithelial cells transit their fate to myofibroblast under certain circumferences in the kidney [33] as well as in the other organs [41], a process called EMT. Diverse origins of myofibroblast have long been debated until a previous report quantitatively concluded that the resident tissue fibroblasts are the major population that contribute to organ fibrosis, as least in the kidney [33]. In this context, our observations from NRK-49F cells (Figure 8) provide evidence that CG directly inhibits activation of fibroblast to myofibroblast under stimulation of Ang II. On the other hand, the effect of CG on the other cells that are known to be involved in renal fibrosis remains inconclusive. Besides the resident fibroblasts and epithelial cells we demonstrated here, endothelial cells [42] and bone marrow-derived cells [43] also transit to myofibroblasts in in vivo models of kidney diseases. The direct anti-fibrotic effect of CG in these cell populations should further be tested.

HDACs deacetylate histones to alter the electrostatic condition of chromatin, suppressing gene expression [44]. Their role has been originally highlighted in nucleosomal proteins as an epigenetic modulator. It is now, however, clear that HDACs also deacetylate many non-histone proteins, such as transcription factors [45,46]. Thus far, 18 mammalian HDACs encoded by distinct genes have been identified and are divided into four main classes according to their sequence homology and expression patterns: class I HDACs (1, 2, 3, and 8), class IIa HDACs (4, 5, 7, and 9), class IIb HDACs (6 and 10), class III HDACs (sirtuin family: sirt 1–7), and class IV HDAC (HDAC11). Although CG inhibits all subtypes of HDACs, the specificity of individual HDAC inhibitors depends on their characteristics [19]. On the basis of a series of recent reports by our group [24,25], we raised several important questions to be answered in future studies, which are also limitations of the present study. First, the specific subtypes of HDAC inhibitors that are enhanced in *Col4a3*^−/−^ mice should be dissected, as the effect of CG cannot discriminate the individual HDAC subtypes. Second, whether or not expression or activity of HDACs are differentially regulated depending on the experimental models should be determined. Finally, specific molecular targets of CG should be clarified by unveiling the site of epigenetic modulation through whole genomic sequencing.

In conclusion, we report that CG is effective both in proximal tubular epithelial cells by inhibiting local RAS and TGFβ signaling activation and in fibroblasts by blocking their transition to myofibroblasts, in order to attenuate renal fibrosis in a murine model of Alport syndrome. Further studies on the clinical application of this novel HDAC inhibitor should be followed to demonstrate its efficacy in human subjects with Alport syndrome.

## 4. Materials and Methods

### 4.1. Experimental Animals and Protocols

The experimental protocol was approved by the Animal Care Regulations Committee of Chonnam National University Medical School (CNU IACUC-H-2017-40, approved on 20 June 2017). Wild-type and *Col4a3*^−/−^ mice [5] on a congenic 129X1/SvJ background were purchased from the Jackson Laboratory (Bar Harbor, ME, USA), and only male mice were used in this study. CG treatment began at 4 weeks of age, and continued for 3 weeks, where CG200745 (Crystal Genomics, Seongnam-si, Gyeonggi-do, Korea) dissolved in drinking water was administered at a dose of 30 mg/kg/day, whereas only vehicle was supplied for the other experimental groups. Mice were sacrificed at 7 weeks of age.

### 4.2. Urine NGAL Measurement by ELISA

Urine samples were collected at the time of sacrifice. Urine samples were centrifuged immediately after collection at 8000× *g* for 5 min at room temperature (RT). NGAL levels were determined with a commercial ELISA kit (R&D Systems, Minneapolis, MN, USA), according to the manufacturer’s instructions.

### 4.3. Histology and Immunohistochemistry

The kidney was placed into 10% neutral buffered formalin (Sigma-Aldrich, St. Louis, MO, USA) for fixation overnight at RT. After brief washing with PBS, the fixed kidney was paraffin-embedded, sectioned into 3 μm thickness, stained, and scanned. Hematoxylin and eosin, periodic acid Schiff, and Masson’s trichrome staining were performed, as previously described [9]. For immunohistochemistry, deparaffinized tissue sections were antigen-retrieved by heating at 100 °C for 15 min in citrate buffer, pH 6.0 (Sigma-Aldrich). Following incubation with blocking buffer (1% (*w*/*v*) bovine serum albumin dissolved in 0.3% (*v*/*v*) Triton-X100 in PBS (0.3% PBS-T)) for 1 h at RT, the section was incubated with primary antibodies diluted in blocking buffer overnight at 4 °C. After brief washing with 0.3% PBS-T three times, sections were incubated with horse radish peroxidase-conjugated secondary antibodies diluted in blocking buffer for 1 h at RT. After brief washing with 0.3% PBS-T three times and with PBS once, sections were incubated in 3,3′-diaminobenzidine reaction solution (Abcam, Cambridge, MA, USA). Following rinsing in tap water, the sections were counter-stained with Meyer’s hematoxylin solution, washed in tap water, dehydrated in ethanol and xylene, and mounted. Primary and secondary antibodies used in immunohistochemistry are listed in Table S1.

### 4.4. Detection of Apoptosis with TUNEL Staining

Apoptosis of tubular epithelial cells was detected with TUNEL staining with ApopTag Plus Peroxidase In Situ Apoptosis Kit (Sigma-Aldrich), according to the manufacturer’s instructions.

### 4.5. Cell Culture

HK-2 cells (American Type Culture Collection, Manassas, VA, USA), an immortalized human kidney proximal tubular epithelial cell line, were cultured in Dulbecco’s modified Eagle’s medium (DMEM) and Ham’s F-12 medium (Sigma-Aldrich) supplemented with 10% fetal bovine serum (FBS; Life Technologies; Gaithersburg, MD, USA), 100 U/mL penicillin, and 100 mg/mL streptomycin (Sigma-Aldrich). NRK-49F cells, a rat kidney interstitial fibroblast cell line (American Type Culture Collection), were cultured in complete DMEM/high glucose media (WelGene, Daegu, Korea) supplemented with 5% FBS, 50 U/mL penicillin, and 50 μg/mL streptomycin. These cells were chosen, because HK-2 [25,47,48] and NRK-49 [49,50,51] cells are the most widely accepted cell lines, representative of proximal tubular epithelial cells and renal interstitial fibroblasts, respectively. Cells were passaged every 3–4 days in 100 mm, were then incubated in a humidified atmosphere of 5% CO_2_ and 95% air at 37 °C for 24 h, and were sub-cultured until 70–80% confluence. For treatment with recombinant proteins or chemicals, cells were plated onto 60 mm dishes and incubated for 24 h with serum-free media for HK-2 cells or with media with 0.5% FBS for NRK-49F cells, prior to treatment with recombinant human Ang II (rhAng II; Bachem, Bubendorf, Switzerland) at a concentration of 1 μM for HK-2 cells and 5 μM for NRK-49F cells for an additional 16 h. CG (10 μM) was added 1 h prior to rhAng II treatment.

### 4.6. Measurement of Reactive Oxygen Species Generation

Level of intracellular and mitochondrial reactive oxygen species were assessed using 5,6-chloromethyl-2′,7′-dichlorodihydrofluorescein diacetate (CM-H2DCFDA; Invitrogen, Carlsbad, CA, USA). Labeling with both probes was conducted on live cells, but not fixed cells. After this, cells were treated with 0.5 mM H_2_O_2_ for 30 min, and then were loaded with 10 μM CM-H2DCFH-DA for 30 min at 37 °C. Images were immediately visualized using a laser scanning microscope (LSM 800; Carl Zeiss, Oberkochen, Germany).

### 4.7. Semi-Quantitative Immunoblotting

The kidney tissue was homogenized in modified RIPA buffer (150 mM sodium chloride, 50 mM Tris-HCl (pH 7.4), 1 mM EDTA, 1% *v*/*v* Triton-X 100, 1% *w*/*v* sodium deoxycholic acid, 0.1% *v*/*v* SDS). Cells were harvested by scrapping the plate in modified RIPA buffer. The tissue homogenates or cell lysates were centrifuged at 4000× *g* for 15 min at 4 °C. Total protein concentration in the supernatants was measured by bicinchoninic acid assay kit (Pierce; Rockford, IL, USA). All samples were adjusted to reach the same final protein concentrations. Then, the supernatant was boiled with loading buffer for 5 min. Proteins in tissue lysates were separated by 6–12% SDS-PAGE gel, and were electrophoretically transferred onto nitrocellulose membranes (Hybond ECL RPN3032D; Amersham Pharmacia Biotech, Little Chalfont, United Kingdom) using Bio-Rad Mini Protean II apparatus (Bio-Rad, Hercules, CA, USA). After incubation with blocking buffer (80 mM Na_2_HPO_4_, 20 mM NaH_2_PO_4_, 100 mM NaCl, and 0.1% Tween-20 at pH 7.5) for 1 h at RT, the membranes were incubated with primary antibodies overnight at 4 °C. Following brief washing, the membranes were then incubated with primary antibodies for 1 h at RT. The immunoblots were detected with an enhanced chemiluminescence system kit (EMD Millipore). Densitometry was calculated by Scion Image software (Scion Corp, Frederick, MD, USA). Primary and secondary antibodies used in immunoblottings are listed in Table S2. The raw data for immunoblotting are listed in Figures S2–S8.

### 4.8. Real-Time qPCR

Kidneys were homogenized in Trizol reagent (Invitrogen, Carlsbad, CA, USA). Cells were harvested by scrapping the plate in the same reagent. RNA was extracted from snap-frozen kidney tissue using the RNeasy Mini kit (Qiagen, Mississauga, Canada), as previously described with minor modification [52]. Briefly, cDNA was reverse-transcribed from 5 μg of total RNA with SuperScript II Reverse Transcriptase (Invitrogen). The relative level of tissue mRNA was determined by real-time qPCR, using a Rotor-Gene 3000 Detector System (Corbette research, Mortlake, New South Wales, Australia). The primers used in real-time qPCR are listed in Table S3.

### 4.9. Statistical Analyses

Unless specified otherwise, results are expressed as mean ± SD. One-way ANOVA with Newman–Keuls multiple comparison test was used for comparison of three groups or more. All statistical analyses were performed with the GraphPad Prism software (GraphPad Software, La Jolla, CA, USA). A *p*-value of less than 0.05 was considered significant.

## Figures and Tables

**Table 1:** Effect of CG200745 (CG) on the renal function in *Col4a3*^−/−^ mice.

	WT	*Col4a3* ^−/−^	*Col4a3*^−/−^+CG
Body weight (g)	24..20 ± 1.65	20.18 ± 1.09 **	20.71 ± 1.17 **
Kidney weight (g)	0.16 ± 0.02	0.18 ± 0.02	0.16 ± 0.01
Kidney weight/body weight (g/kg)	6.40 ± 0.82	8.79 ± 0.39 **	7.61 ± 0.14 *^, #^
Urine NGAL (ng/mL)	61.1 ± 7.63	848.80 ± 25.56 **	408.10 ± 150.10 **^, ##^

Values are expressed as mean ± SD. NGAL, neutrophil gelatinase-associated lipocalin. *p < 0.05, **p < 0.01 vs. WT mice; ^#^*p* < 0.05, ^##^*p* < 0.01 vs. *Col4a3*^−/−^ mice by one-way ANOVA with Newman–Keuls multiple comparison test.

## Supplementary Materials

Supplementary materials can be found at https://www.mdpi.com/1422-0067/21/4/1473/s1. Table S1: List of primary and secondary antibodies for immunohistochemistry. Table S2: List of primary and secondary antibodies for immunoblotting. Table S3: List of primer sequences for real-time qPCR. Figure S1. CG alone did not induce apoptosis in HK-2 cells. Figure S2. Raw data for immunoblotting related to Figure 2. Figure S3. Raw data for immunoblotting related to Figure 3. Figure S4. Raw data for immunoblotting related to Figure 4. Figure S5. Raw data for immunoblotting related to Figure 5. Figure S6. Raw data for immunoblotting related to Figure 6. Figure S7. Raw data for immunoblotting related to Figure 7. Figure S8. Raw data for immunoblotting related to Figure 8.
