# Supplementary material for: CG200745, a Novel HDAC Inhibitor, Attenuates Kidney Fibrosis in a Murine Model of Alport Syndrome
Source: Int J Mol Sci. 2020 Feb 21;21(4):1473. doi: 10.3390/ijms21041473 (PMC7073208; doi:10.3390/ijms21041473)
Supplement: Supplementary file 1 [file ijms-21-01473-s001.pdf]

CG200745, a novel HDAC inhibitor, attenuates kidney fibrosis in a murine model of Alport syndrome

Sang Heon Suh[1,2], Hong Sang Choi[1,2], Chang Seong Kim[1,2], In Jin Kim[1], Hyunju Cha[3], Joong Myung Cho[3], Seong Kwon Ma[1,2], Soo Wan Kim[1,2,*], and Eun Hui Bae[1,2,*]

[1]Department of Internal Medicine, Chonnam National University Medical School, Gwangju 61469, Korea; [2]Department of Internal Medicine, Chonnam National University Hospital, Gwangju 61469, Korea;[3]Crystal Genomics, Inc., 5 F, Bldg A, Korea Bio Park, Seongnam 13488, Korea

*These authors have contributed equally to this manuscript as correspondence authors.

Table of Contents

Table S1. List of primary and secondary antibodies for immunohistochemistry Page 2
Table S2. List of primary and secondary antibodies for immunoblotting Page 3
Table S3. List of primer sequences for real-time qPCR Page 4
Figure S1. CG alone does not induce apoptosis in HK-2 cells. Page 5
Figure S2. Raw data for immunoblotting related Figure 2 Page 6
Figure S3. Raw data for immunoblotting related Figure 3 Page 7
Figure S4. Raw data for immunoblotting related Figure 4 Page 8
Figure S5. Raw data for immunoblotting related Figure 5 Page 9
Figure S6. Raw data for immunoblotting related Figure 6 Page 10
Figure S7. Raw data for immunoblotting related Figure 7 Page 11
Figure S8. Raw data for immunoblotting related Figure 8 Page 12

Table S1. List of primary and secondary antibodies for immunohistochemistry

	Host	Reactivity	Supplier	Cat. No.
F4/80	Rat	Mouse	Bio-rad	MCA497GA
Transforming growth factor β1	Rabbit	Human, Mouse	Abcam	ab92486
α smooth muscle actin	Mouse	Human, Mouse	Sigma-Aldrich	A3854
Rabbit IgG, HRP-linked	Goat	Rabbit IgG	Vector	PI-1000
Rat IgG, HRP-linked	Goat	Rat IgG	Vector	PI-9400
Mouse IgG, HRP-linked	Goat	Mouse IgG	Vector	PI-2000

Table S2. List of primary and secondary antibodies for immunoblotting.

	Host	Reactivity	Supplier	Cat. No.
Angiotensin-converting enzyme	Goat	Human, Mouse	Santa Cruz	sc12187
Angiotensin-converting enzyme 2	Goat	Mouse	R&D	AF3437
Angiotensin-converting enzyme 2	Rabbit	Human	Cell Signaling	#4355
Angiotensin II-III	Mouse	Human, Mouse	Novus Biologicals	NB100-62346
Ang II type 1 receptor	Rabbit	Human, Mouse	Santa Cruz	sc-1173
Ang II type 2 receptor	Rabbit	Human, Mouse	Santa Cruz	sc-9040
BAX	Rabbit	Human, Mouse	Cell signaling	#2772
BCL2	Rabbit	Human, Mouse	Cell signaling	#3498
CD68	Rabbit	Mouse	Abcam	ab31630
Caspase 3	Rabbit	Human, Mouse	Cell signaling	#9662
Cleaved caspase 3	Rabbit	Human, Mouse	Cell signaling	#9661
ERK1/2	Rabbit	Human, Mouse	Cell signaling	#9102
Fibronectin	Rabbit	Human, Mouse, Rat	Abcam	ab2413
Heme oxygenase 1	Mouse	Mouse	Abcam	ab13248
JNK	Rabbit	Human, Mouse	Cell signaling	#9252
P38	Rabbit	Human, Mouse	Cell signaling	#9212
Phopho-ERK1/2	Rabbit	Human, Mouse	Cell signaling	#9101
Phopho JNK	Rabbit	Human, Mouse	Cell signaling	#9251
Phospho P38	Rabbit	Human, Mouse	Cell signaling	#9215
Phospho SMAD2/3	Rabbit	Human, Mouse, Rat	Cell Signaling	#8828
SMAD4	Rabbit	Human, Mouse, Rat	Cell Signaling	#38454
SMAD2/3	Rabbit	Human, Mouse, Rat	Cell Signaling	#3102
TNFα-converting enzyme (TACE)	Rabbit	Human, Mouse	Millipore	AB19027
Transforming growth factor β	Rabbit	Human, Mouse, Rat	Cell Signaling	#3711
α smooth muscle actin	Mouse	Human, Mouse, Rat	Sigma-Aldrich	A3854
β-actin	Rabbit	Human, Mouse, Rat	Cell Signaling	#3711
Goat IgG, HRP-linked	Rabbit	Goat IgG	Sigma-Aldrich	AP106P
Rabbit IgG, HRP-linked	Goat	Rabbit IgG	Cell Signaling	#7074
Mouse IgG, HRP-linked	Horse	Mouse IgG	Cell Signaling	#7076

Table S3. List of primer sequences for real-time qPCR

	Forward	Reverse
Homo sapience		
GAPDH	GACATCAAGAAGGTGGTGAA	TGTCATACCAGGAAATGAGC
TGFB1	CAGAAATACAGCAACAATTCCTGG	TTGCAGTGTGTTATCCCTGCTGTC
Rattus norvegicus		
Acta2 (αSMA)	TGTGCTGGACTCTGGAGATG	GAAGGAATAGCCACGCTCAG
Col1a1 (collagen, type I)	CAACCTCAAGAAGTCCCTGC	ACAAGCGTGCTGTAGGTGAA
Gapdh	ATCAAATGGGGTGATGCTGGTGCTG	CAGGTTTCTCCAGGCGGCATGTCAG
Fn1 (fibronectin)	CATGAAGGGGGTCAGTCCTA	GTCCATTCCCCTTTTCCATT
Mus musculus		
Acta2 (αSMA)	ACTGGGACGACATGGAAAAG	CATCTCCAGAGTCCAGCACA
Col1a1 (collagen, type I)	GAGCGGAGAGTACTGGATCG	TACTCGAACGGGAATCCATC
Gapdh	TGTGTCCGTCGTGGATCTGA	GATGCCTGCTTCACCACCTT
Icam1	AACTTTTCAGCTCCGGTCCTG	TCAGTGTGAATTGGACCTGCG
Il-6	ACAACCACGGCCTTCCCTACTT	CACGATTTCCCAGAGAACATGTG
Fn1 (fibronectin)	ACACGGTTTCCCATTACGCCAT	AATGACCACTGCCAAAGCCCAA
Tgfb1 (TGFβ)	CAACAATTCCTGGCGTTACCTTGG	GAAAGCCCTGTATTCCGTCTCCTT
Tnf (TNFα)	GCATGATCCGCGACGTGGAA	AGATCCATGCCGTTGGCCAG
Vcam1	TCTCTCAGGAAATGCCACCC	CACAGCCAATAGCAGCACAC

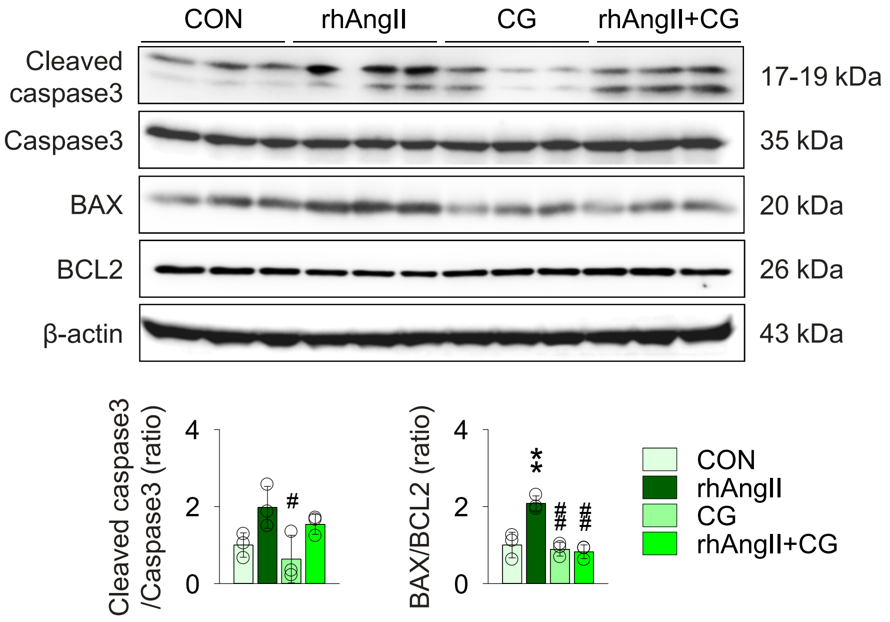

Figure S1. CG alone does not induce apoptosis in HK-2 cells. Comparison of protein expression level for molecules related to apoptosis determined by immunoblotting in HK-2 cells after stimulation with vehicles or recombinant human Ang II (rhAng II) with or without co-treatment of CG (n = 3/group). **P < 0.01 vs. control cells (CON); $^{\#}$P < 0.05, $^{\#\#}$P < 0.01 vs. rhAng II-treated cells by one-way ANOVA with Newman-Keuls multiple comparison test.

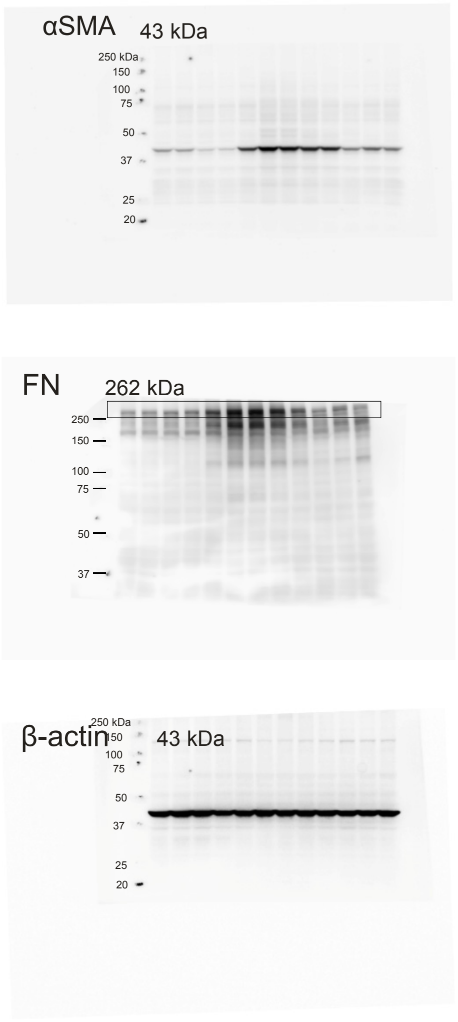

Figure S2. Raw data for immunoblotting related Figure 2

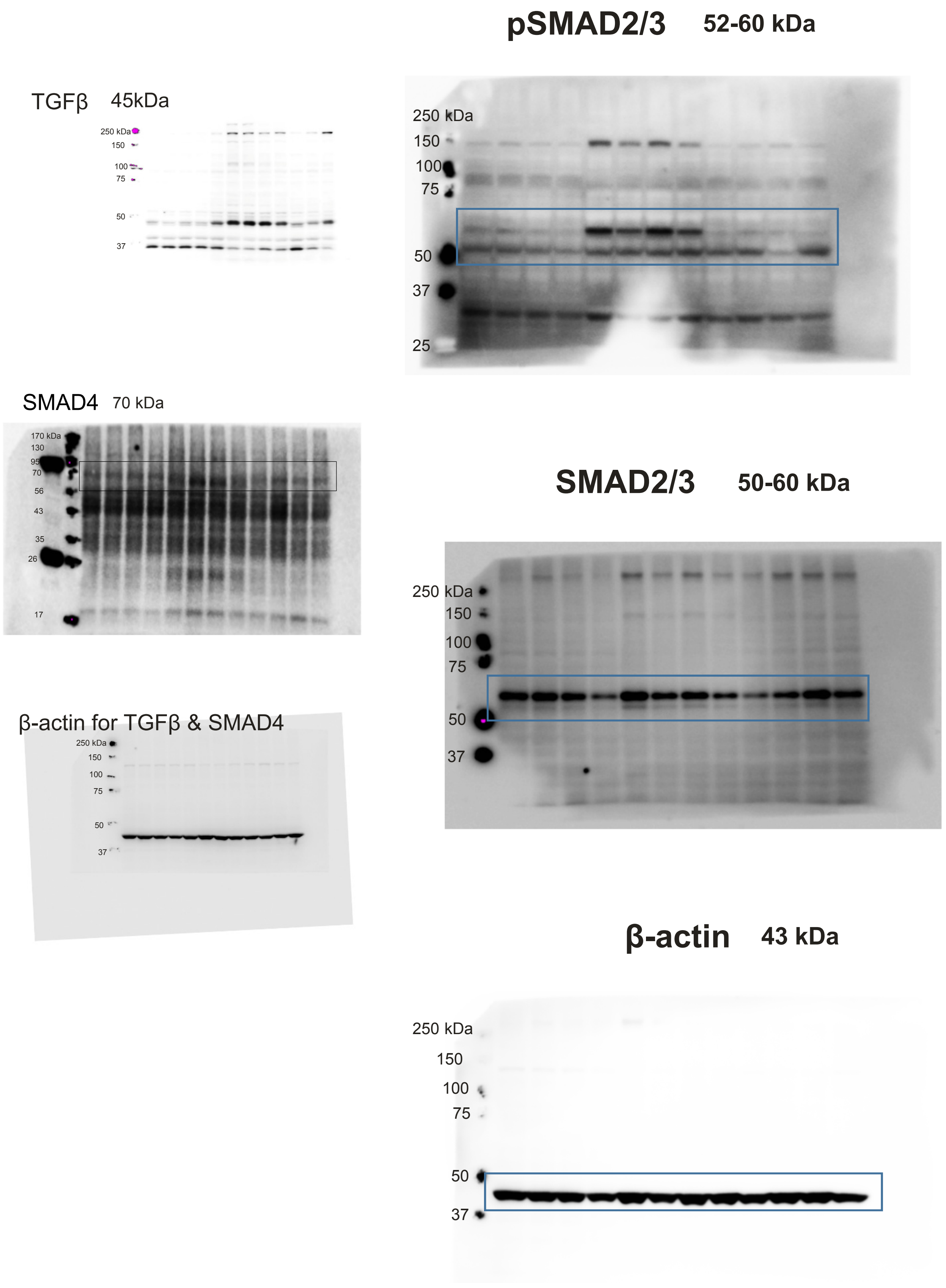

Figure S3. Raw data for immunoblotting related Figure 3

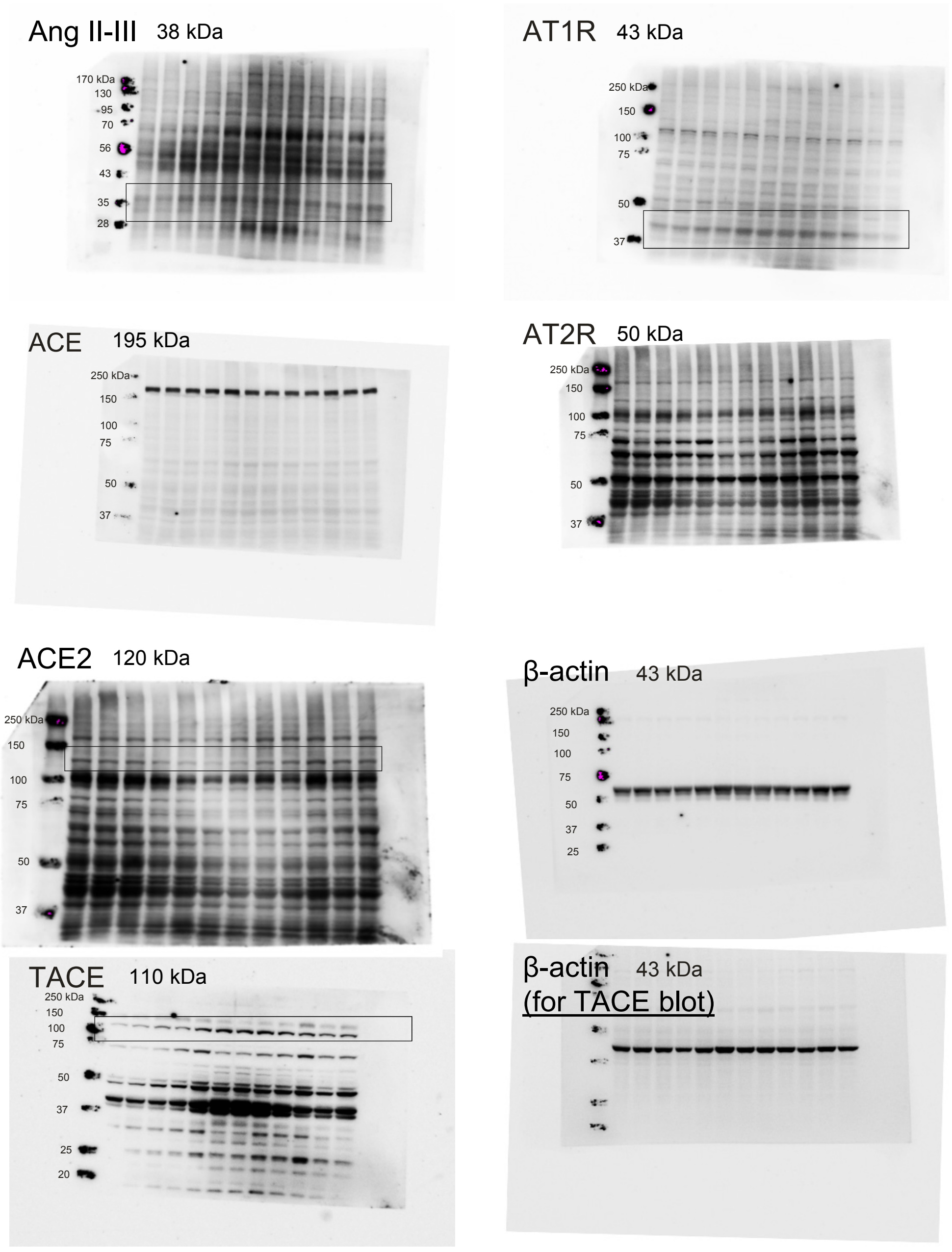

Figure S4. Raw data for immunoblotting related Figure 4

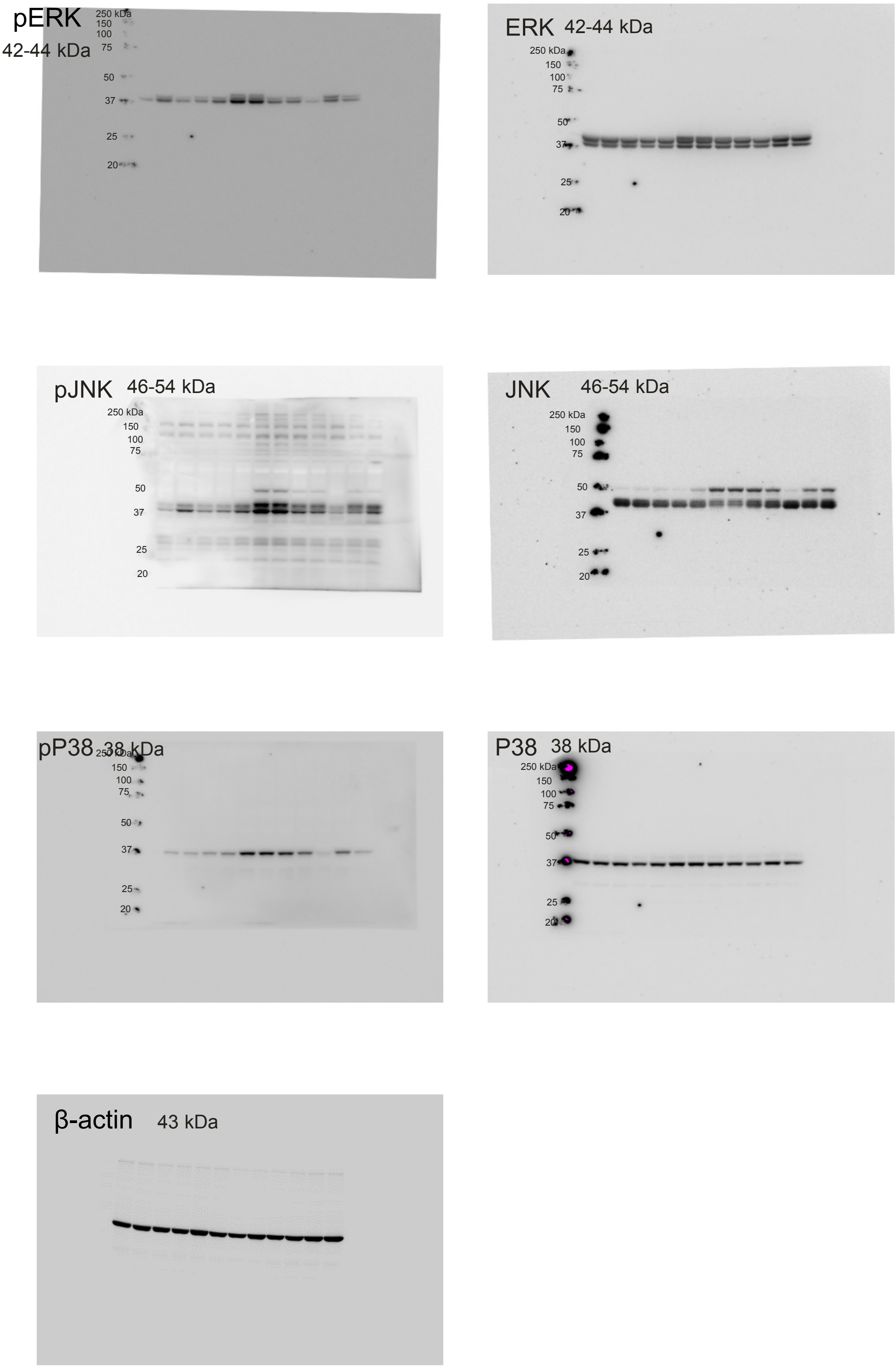

Figure S5. Raw data for immunoblotting related Figure 5

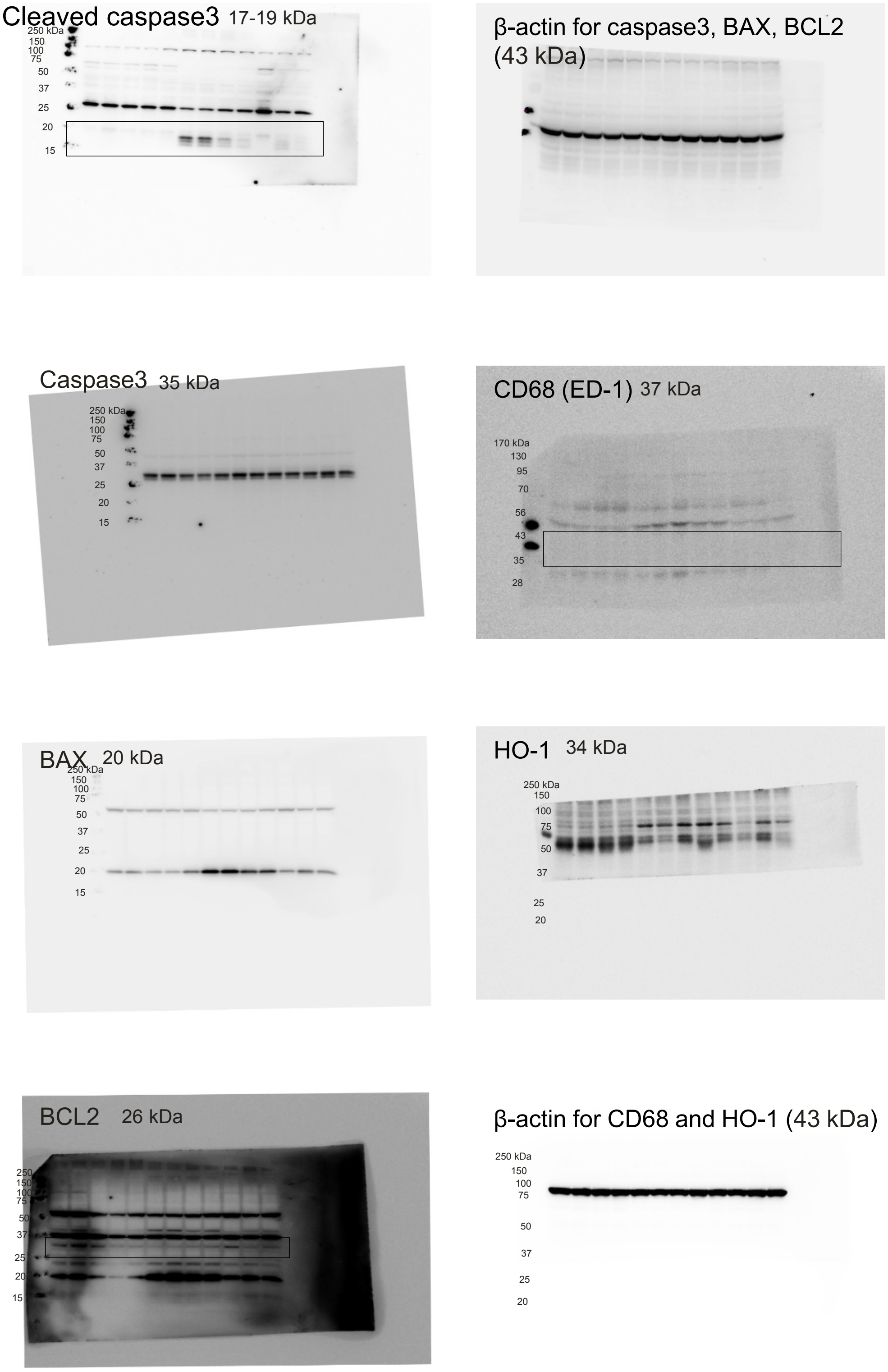

Figure S6. Raw data for immunoblotting related Figure 6

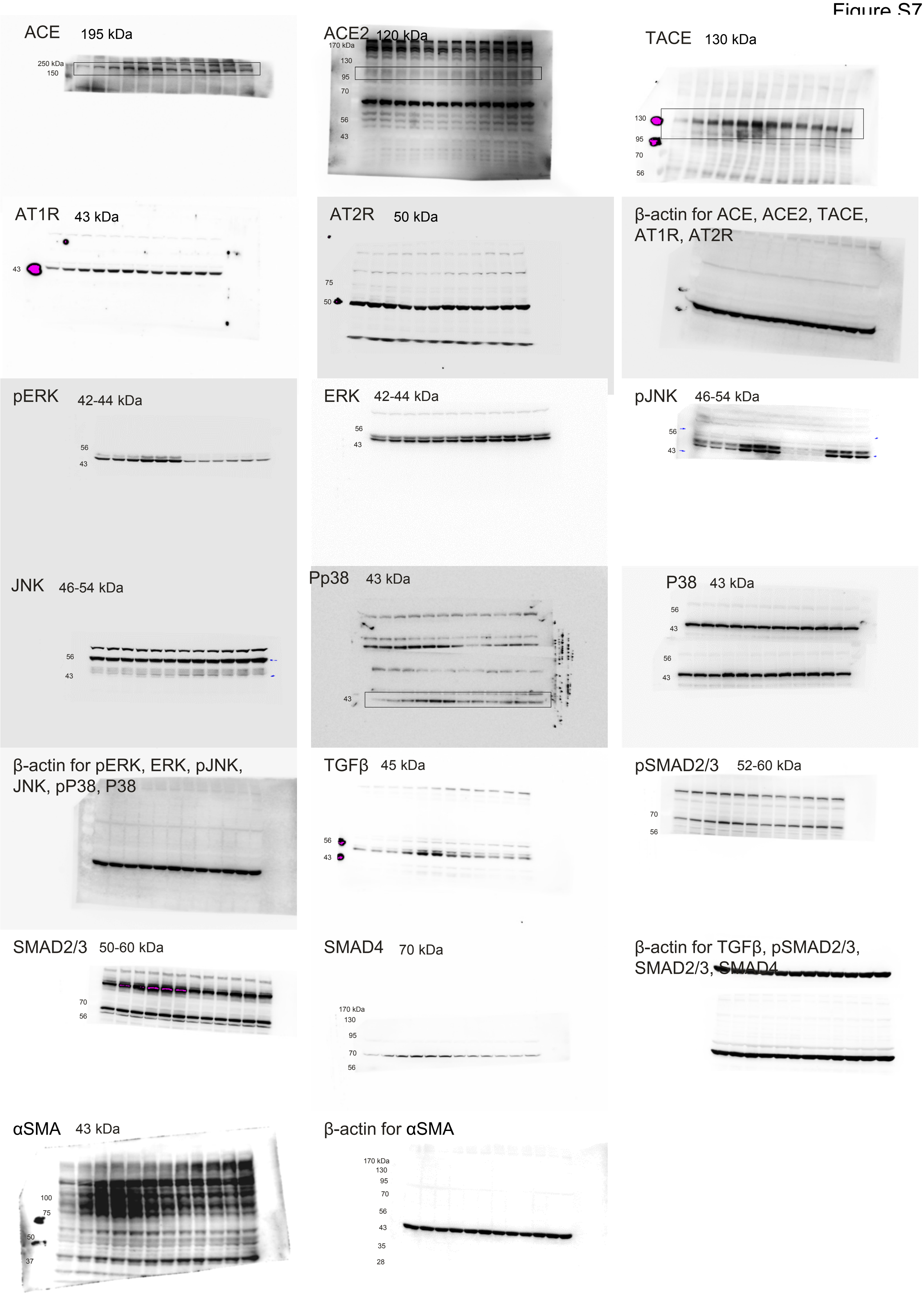

Figure S7. Raw data for immunoblotting related Figure 7

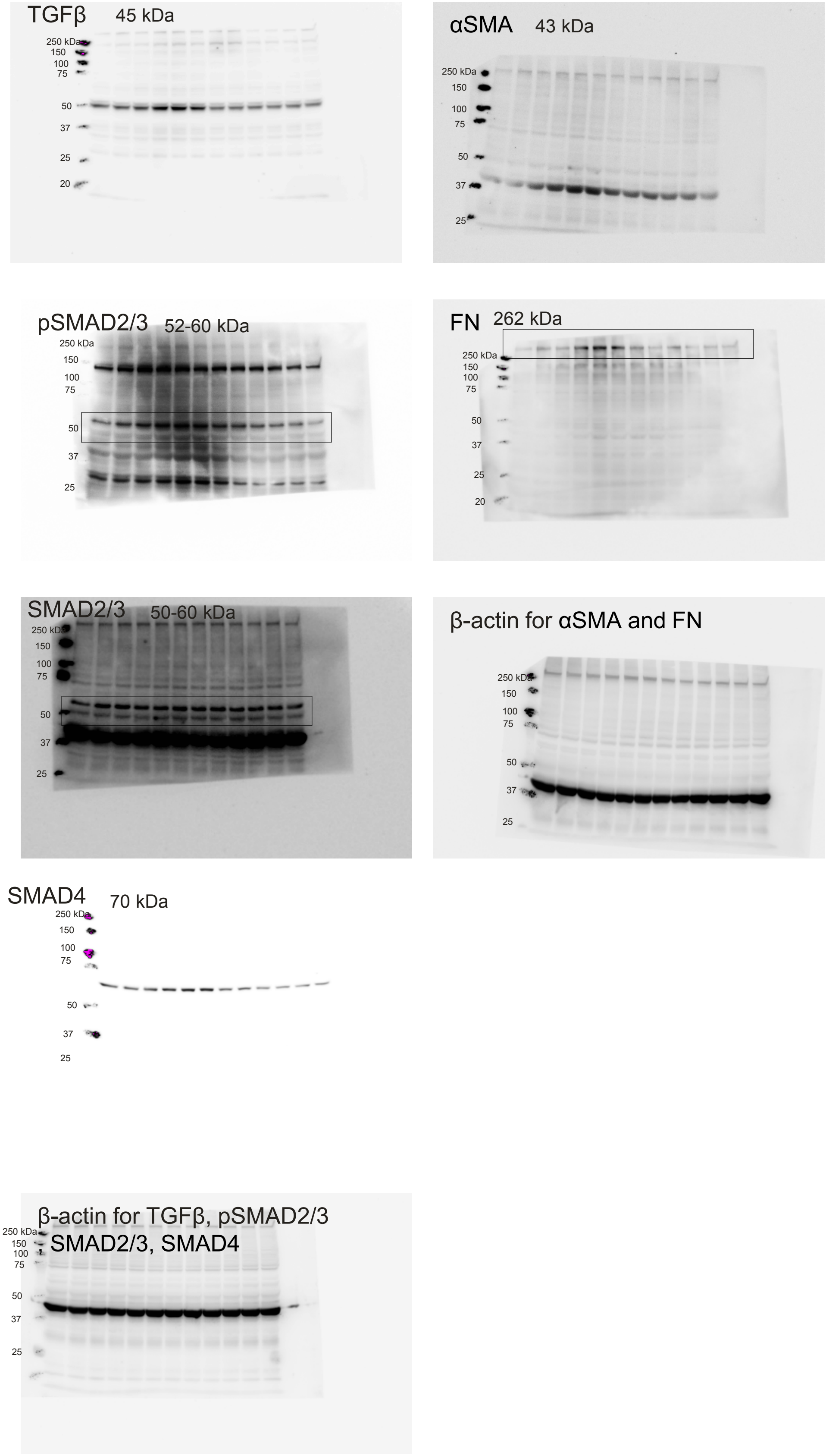

Figure S8. Raw data for immunoblotting related Figure 8
